# Early infant diagnosis of HIV-1 infection in Luanda, Angola, using a new DNA PCR assay and dried blood spots

**DOI:** 10.1371/journal.pone.0181352

**Published:** 2017-07-17

**Authors:** Francisco Martin, Claudia Palladino, Rita Mateus, Anna Bolzan, Perpétua Gomes, José Brito, Ana Patrícia Carvalho, Yolanda Cardoso, Cristovão Domingos, Vanda Sofia Lôa Clemente, Nuno Taveira

**Affiliations:** 1 Research Institute for Medicines (iMed.Ulisboa), Faculdade de Farmácia, Universidade de Lisboa, Lisbon, Portugal; 2 Hospital da Divina Providência, Luanda, Angola; 3 Laboratório de Microbiologia Clínica e Biologia Molecular, Serviço de Patologia Clínica, Centro Hospitalar de Lisboa Ocidental, E.P.E., Lisbon, Portugal; 4 Centro de Investigação Interdisciplinar Egas Moniz, Instituto Superior de Ciências da Saúde Egas Moniz, Caparica, Portugal; 5 Ministério da Saúde de Angola - Instituto Nacional de Saúde Pública (INSP), Luanda, Angola; 6 Ministério da Saúde de Angola - Instituto Nacional de Luta contra a Sida (INLS), Luanda, Angola; Institut Pasteur of Shanghai Chinese Academy of Sciences, CHINA

## Abstract

**Background:**

Early diagnosis and treatment reduces HIV-1-related mortality, morbidity and size of viral reservoirs in infants infected perinatally. Commercial molecular tests enable the early diagnosis of infection in infants but the high cost and low sensitivity with dried blood spots (DBS) limit their use in sub-Saharan Africa.

**Objectives:**

To develop and validate a sensitive and cheap qualitative proviral DNA PCR-based assay for early infant diagnosis (EID) in HIV-1-exposed infants using DBS samples.

**Study design:**

Chelex-based method was used to extract DNA from DBS samples followed by a nested PCR assay using primers for the HIV-1 integrase gene. Limit of detection (LoD) was determined by Probit regression using limiting dilutions of newly produced recombinant plasmids with the integrase gene of all HIV-1 subtypes and ACH-2 cells. Clinical sensitivity and specificity were evaluated on 100 HIV-1 infected adults; 5 infected infants; 50 healthy volunteers; 139 HIV-1-exposed infants of the Angolan Pediatric HIV Cohort (APEHC) with serology at 18 months of life.

**Results:**

All subtypes and CRF02_AG were amplified with a LoD of 14 copies. HIV-1 infection in infants was detected at month 1 of life. Sensitivity rate in adults varied with viral load, while diagnostic specificity was 100%. The percentage of HIV-1 MTCT cases between January 2012 and October 2014 was 2.2%. The cost per test was 8-10 USD which is 2- to 4-fold lower in comparison to commercial assays.

**Conclusions:**

The new PCR assay enables early and accurate EID. The simplicity and low-cost of the assay make it suitable for generalized implementation in Angola and other resource-constrained countries.

## Introduction

HIV-1 mother-to-child-transmission (MTCT) is the main mode of infection among the pediatric population and is disproportionately affecting children in impoverished countries. Despite the decline in MTCT rate in recent years in most of the sub-Saharan Africa, it is estimated that 150,000 children became newly infected with HIV in 2015 [1]. Children infected perinatally are at high risk of rapid disease progression and death during the first year of life without antiretroviral therapy (ART) [2]. Given the reported benefits of early ART initiation in reducing HIV-1-related mortality and long-term morbidity [3] and reducing the size of the HIV-1 reservoirs [4], early HIV-1 diagnosis in newborns represents the critical gateway to timely initiation of life-saving ART. Serological assays do not permit the early diagnosis of HIV-1 infection because of the persistence of maternal HIV-1 antibodies in infants during the first 12-18 months of life. The WHO recommends the use of molecular-based virological testing to determine the infection status for HIV-1-exposed infants during the first 4-6 weeks of life or at the earliest opportunity thereafter [5]. Despite the high accuracy of tests which detect HIV RNA or p24, their sensitivity could potentially be affected in settings of expanded ART for prevention of MTCT (option B and B+), which reduce circulating HIV-1 RNA and viral particles [6]. Qualitative DNA PCR test which detect proviral DNA in peripheral blood mononuclear cells (PBMCs) is recommended for early infant diagnosis (EID) of HIV-1 and is the most widely implemented test in resource-limited settings [7–8]. The considerable uptake of HIV-1 DNA molecular tests is driven by the lower costs compared with quantitative assays along with their good sensitivity when performed on blood microsamples or dried blood spots (DBS) [7]. The use of DBS presents several advantages such as reduced costs for collection, storage and shipping. Thus, DBS samples are convenient for increasing access to testing in settings with poor healthcare provision and referral laboratories [9]. Currently, two HIV-1 DNA assays are commercially available for EID using DBS: the COBAS® AmpliPrep/COBAS® TaqMan® HIV-1 Qualitative Test (Roche Molecular System Inc., Branchburg, NJ), which recently replaced the Roche Amplicor® DNA test v1.5, and the RealTi*m*e HIV-1 Qualitative Test (Abbott Molecular, Des Plaines, IL) [8]. These assays are highly sensitive but require sophisticated instrument platforms whose cost with related equipment (e.g. centrifuge; biosafety cabinet; freezer) can range from about US$ 100,000 to more than US$ 200,000. Recently, innovative technologies designed for use at or near the point-of-care (such as the Cepheid Xpert® HIV-1 Qual assay and the Alere™ q HIV-1/2 Detect) have been developed and the Xpert® HIV-1 Qual assay has been validated for both whole blood and DBS specimens [8].

Despite the recent advances in prevention of MTCT, Angola reported one of the highest rates of MTCT (25%) among the 22 priority countries included in the UNAIDS global plan [1]. The EID national program implemented in 2007 based on the Roche Amplicor^®^ DNA test v1.5 was interrupted in 2012; consequently the coverage of virological testing for infants is currently very low and only 14% of HIV-1-exposed newborns received virological HIV-1 testing within the first 2 months of life in 2014 [1]. Several challenges may prevent the implementation of molecular diagnostic tests such as the high cost of available commercial PCR assays and their limited sensitivity with highly divergent HIV-1 subtypes which co-circulate in the country [10–12].

In this article, we developed and validated a new qualitative HIV-1 DNA PCR assay for the early infant diagnosis of HIV-1 infection in Angola and other countries with similar complex epidemics. Using this highly sensitive and specific assay we identified the new cases of MTCT of HIV-1 in the APEHC pediatric cohort recently established in a major hospital in Luanda.

## Participants and methods

### Study design

This assay validation study describes the use of DBS to diagnose HIV-1 in infants born to HIV-infected mothers in Angola between January 2012 and October 2014. The STROBE checklist was used to help design and conduct the study [13].

### Ethics statement

This study was conducted according to the Declaration of Helsinki with the approval of the National Ethical Committee of Angola and the Ethic Committee of the *Centro Hospitalar de Lisboa Ocidental*, *E*.*P*.*E* in Portugal. Written informed consent was obtained from all participants or from parents/guardians for their infants and from HIV-1 seronegative healthy volunteers.

### The Angolan Pediatric HIV Cohort and clinical samples

The Angolan Pediatric HIV Cohort (APEHC) has prospectively collected data on HIV-1-infected pregnant women and their infants attending the municipal *Hospital da Divina Providência* (HDP) since March 2012 (S1 Data set file). HDP is located in the Luanda district, which is the most populated district of Luanda city, Angola. Epidemiological, clinical and laboratory data were collected at study entry and every 6 months thereafter for women. Infants were followed according to the perinatal care service offered at the HDP, which includes clinical and biological examination at months 1, 3, 6, and 18 of life. No specific recommendations for HIV treatment was made for women enrolled in the cohort and physicians followed the WHO guidelines for prevention of MTCT [7]. Infants received NVP once daily from birth through age 4-6 weeks in accordance with option B [7]. HIV-1 testing for both mothers and newborns was free of charge and was performed using two rapid tests for detection of antibodies against HIV-1/2 (Determine HIV ½ and Uni-Gold HIV) as recommended by WHO [14]. The diagnosis required consistent results of the two different tests. An infant was considered as infected if anti-HIV-1 antibodies were detected on two separate samples collected at least three months apart and persisted after 18 months of age; it was considered non-infected if serological testing was negative on two separate samples before or after 18 months. Undetermined cases were those with discordant results between the two rapid tests and were further tested using an ELISA assay (Vironostika HIV Uni-form II Ag/Ab ELISA test; bioMérieux, France). DBS specimens were obtained from all infants born between January 2012 and October 2014 from HIV-1 infected women enrolled in the APEHC. Additionally, five DBS samples from HIV-1 infected infants aged 2- to 12-days old were obtained from the *Instituto Nacional de Saúde Pública* (INSP). DBS samples were also collected from six women attending the HDP with indeterminate HIV serologic testing results. DBS samples were prepared by spotting 125 μL of whole blood, collected by heel prick for infants and by finger-prick for adults, onto filter paper cards (Whatman® Human ID Blood Stain Cards BFC 180). DBS were dried over night at room temperature, individually enveloped in Glassine paper, inserted in a zip-lock polyethylene bag (Deltalab S.L., Spain) with silica desiccant and stored at -20°C. DBS were subsequently shipped at room temperature to our laboratory at the University of Lisbon for testing.

Two sets of samples were used as clinical controls. One-hundred DBS specimens were collected during 2014 from adults attending a central hospital in Lisbon (*Centro Hospitalar de Lisboa Ocidental*, *E*.*P*.*E*.), who had a confirmed HIV-1 diagnosis done by a 4th generation assay followed by an immunoblot assay on two separate samples. Plasma HIV-1 RNA level was determined in these patients by COBAS® AmpliPrep/COBAS® TaqMan® HIV-1 Test, v2.0 (Roche Diagnostic Systems) with a lower limit of detection of 20 copies/mL. CD4^+^ T-lymphocytes were quantified by flow cytometry. Fifty DBS collected from HIV-1 seronegative healthy volunteers were used as negative controls.

To further test the diagnostic specificity of the assay, samples obtained from patients infected with HBV, HCV and CMV were also analyzed. These samples were collected from HIV-1 seronegative adults attending the *Centro Hospitalar de Lisboa Ocidental*, *E*.*P*.*E*. who had a confirmed diagnosis of hepatitis B (HBV) done by detection of hepatitis B surface antigen, HBsAg, (N = 10), or hepatitis C (HCV) done by detection of antibodies to HCV and viral RNA (N = 10), or cytomegalovirus (CMV) done by detection of antibodies to CMV (N = 10).

### Production of DBS samples with control plasmids

A 1553 bp pol gene product comprising the highly conserved IN gene region was amplified from the reference subtype B HIV-1 SG3.1 [15] and from nine primary viral isolates belonging to the most common HIV-1 clades circulating in Angola S1 Table by RT-PCR as described previously [10, 16–18]. Primers for this PCR were designed using PerlPrimer® v1.1.21 software and reference sequences present in the Los Alamos HIV sequence database (http://www.hiv.lanl.gov). Their sequence and location in the HIV-1 genome are shown in S2 Table. PCR products were cloned into pcDNA 3.1D/V5-His-TOPO plasmid using the protocol indicated by the manufacturer (Invitrogene Corp., Carlsbad, CA) and sequenced by the Sanger method. Phylogenetic analysis was used to confirm the subtype of the isolates (data not shown). Purified control plasmids were quantified by spectroscopy at 260 nm using a calibration curve and then serially diluted in HIV-1 seronegative blood and spotted (125 μL) onto Human ID blood stain cards to obtain a concentration of 50 to 5,000 copies/DBS.

### Production of DBS samples with ACH-2 cells

ACH-2 cells were used as analytical control for DNA PCR assays as they contain a single, integrated HIV-1 subtype B proviral DNA per cell [19–20]. ACH-2 cells were diluted in 125 μL of HIV seronegative blood (5 log serial dilutions) and spotted on Human ID blood stain cards to obtain a DBS control panel containing 50-5,000 cells.

### Chelex DNA extraction method

DNA was extracted from DBS samples using the polyvalent cationic resin Chelex 100® (Bio-Rad Laboratories, Hercules, CA, USA). Briefly, six circles of 5 mm diameter were punched from each DBS spot into a 1.5 mL tube. After 30 min washing with sterile water and 3 min centrifugation (15,400 g), 250 μL of 5% Chelex solution was added to the pellet and samples were incubated at 56°C for 15 minutes. The samples were vortexed and centrifuged (15,400 g) for 3 min, prior to a final incubation at 100°C for 8 minutes. Finally, samples were centrifuged and stored at -20°C until the PCR reaction was performed.

### PCR amplification of proviral HIV-1 DNA

A nested PCR was used to amplify a 194 bp fragment of the IN gene. First- and second-round amplifications were performed using the same reaction and cycling conditions. The 25 μL reaction volume contained 1X NH4 buffer, 3 mmol/L MgCl2, 0.5 μmol/L of each dNTP, 0.3 μmol/L forward and reverse primers S2 Table, 1U of Taq DNA polymerase (Bioline® Reagents Ltd, London, UK) plus 2.5 μL of DNA solution from the clinical or control samples (first-round PCR). For the second-round PCR reaction we used 2.5ul of the first-round PCR reaction. Amplification cycling conditions were as follows: denaturation step of 94°C/3 min followed by 35 cycles of denaturation at 94°C/ 45 sec, annealing at 56°C/35 sec, extension at 72°C/ 1 min followed by a single final extension step at 72°C/ 15 min. Amplified products were visualized with green safe staining after electrophoresis in 2% agarose gel. In all cases the human gene C-C chemokine receptor 5 (CCR5) was used as an internal control to confirm the presence and quality of genomic DNA. CCR5 was amplified using primers CCR5c and CCR5d [21] and the cycling conditions used for the amplification of the IN gene region.

### Limit of detection (LoD)

DNA extracted from DBS samples spotted with serial dilutions of control plasmids or ACH-2 cells was subjected to the nested PCR protocol described above. Each template was amplified ≥10 times and the LoD for each subtype and its 95% fiducial confidence interval were estimated by probit regression analysis.

## Results

### Analytical sensitivity

The LoD of the assay was determined by probit regression analysis with DBS spiked with serial dilutions of control plasmids containing the IN gene regions from HIV-1 subtypes A, C, D, F, G, H, J and CRF02_AG. For HIV-1 subtype B, DBS spiked with increasing number of ACH-2 cells, which contain one integrated proviral DNA copy per cell, were used. When the subtype was included in the probit model as an independent factor, the parallelism test chi-square was significant (χ2 = 64.7; df = 8; p < 0.001) which rejects the assumption of equal slopes across subtypes. Therefore, the probit analysis was implemented separately for each subtype. Under these conditions the LoD of the assay varied between 4.3 and 14.4 copies depending on subtype Table 1.

**Table 1:** Limit of detection of the assay for different HIV-1 subtypes as determined using probit regression analysis.

Subtype	Probit results*		
	Significance of Pearson χ^2^ goodness of fit test	LoD (copies/PCR)	95% Confidence Interval
A1	0.330	5.0	4.1 – 8.4
H	0.300	10.1	7.9 – 19.7
B	0.885	9.3	5.0 – 33.1
F	0.914	10.4	7.7 – 27.2
G	0.997	11.8	9.6 – 22.6
J	0.267	4.3	3.3 – 6.3
CRF02_AG	0.252	4.4	3.4 – 6.4
C	0.077	5.5	4.7 – 31.0
D	0.624	14.4	10.4 – 25.2

* Determined in DBS spiked with serial dilutions of control plasmids except for subtype B that was determined in DBS spiked with ACH-2 cells (S1 Fig and S3 Table).

One hundred and twenty five μl of infant’s blood, which is the amount present in each DBS, has 0.4-0.6x10^6^ PBMCs [22]. HIV-1 infected infants harbor an estimate of 13,000 to 75,400 HIV proviral copies per 10^6^ PBMCs [23]. In each PCR reaction, we used 1/100 of the extracted DNA solution (2.5 out of 250 μl); considering only the lower limit of PBMCs that the infants may have in this amount of blood this corresponds to 0.4x10^4^ PBMCs per reaction. Considering the lower number of proviral copies that the HIV-1 infected infants may have in this number of cells this corresponds to a minimum of 52 HIV-1 proviral copies. This is more than 11-fold higher than the lower LoD of our PCR assay assuring that it has high enough sensitivity to detect HIV-1 infection in all infected infants.

### Diagnostic sensitivity and specificity

Diagnostic specificity of the EID assay was 100% since all the HIV-1 seronegative samples tested (n = 186, 50 adults from Portugal and 136 infant DBS samples from the APEHC cohort) were negative for the presence of HIV-1 proviral DNA. This was further confirmed using 30 samples from patients infected with HBV, HCV or CMV as all gave negative results using the new PCR assay S2 Fig.

The clinical sensitivity of the assay was determined with DBS samples collected from 100 HIV-1 infected adult patients from Portugal (patients with chronic infection), from 5 confirmed HIV-1 positive infants obtained from the *Instituto Nacional de Luta contra a Sida* (INLS), Luanda, Angola (infection in these patients was confirmed by serology at month 18 of life and by detection of HIV-1 DNA using the Nuclisens EasyMag/EasyQ, Biomérieux) and from the infants of the APEHC cohort (S1 Data set file). Regarding the Portuguese adult patients, the median CD4 count was 608 cells/mm^3^ (min-max:83-2,075). Nine subjects were severely immunosuppressed (<200 cells/mm^3^), 35 had CD4 count of 200-499 cells/mm^3^, and 52 had ≥500 cells/mm^3^. HIV-1 proviral DNA was detected in 14.3%, 56.3% and 85.7% of the patients with plasma viral load of <20 copies/mL, 20-1,000 copies/mL and >1,000 copies/mL, respectively Table 2. All five HIV-1 infected infants from the INLS were HIV-1 DNA positive by our assay.

**Table 2:** Performance of the new PCR assay in HIV-1-infected adults from Portugal.

EID assay	Adult HIV-1-infected patients (N = 100)			Total
	Undetectable viral load (<20 copies/mL)	Viral load of 20-1,000 copies/mL	Viral load of >1,000 copies/mL	
Positive	11	9	6	26
Negative	66	7	1	74
Total	77	16	7	100
Percentage of detection	14.3	56.3	85.7	--

A total of 154 HIV-1-exposed infants were enrolled in the APEHC cohort and one DBS card containing 4 blood spots per infant was available for the lab tests. The median age was 1 month: 83% (129/154) of infants were 1 month of age, 7% (11/154) were 2-5 months of age, and 9% (14/154) were 6-12 months of age; 50% were girls (n = 77). For the specificity and sensitivity analyses, 15 patients were excluded as follows: 11 infants dropped-out from routine clinical care and 4 infants died before the serology results were confirmed. Those patients were all negative according to our assay. Three out of the 139 samples that were analyzed by our assay were HIV-1 DNA positive; infection with HIV-1 in these infants was confirmed by serology at month 18 (Table 3 and S3 Fig).

**Table 3:** Sensitivity and specificity of the new assay for early infant diagnosis of HIV-1 infection in Angola.

	Infants with HIV-1 serology at month 18 and/or HIV-1 DNA test (N = 144)*	
Our HIV-1 DNA PCR assay	Positive	Negative
Positive	8	0
Negative	0	136
Total	8	136
Sensitivity	100.0%	
Specificity	100.0%	

* Infants from the APEHC cohort (N = 139) plus infants (N = 5) with HIV-1 infection confirmed at the INLS in Luanda, Angola.

All 136 infants with negative results with our assay were HIV-1 seronegative at month 18. Therefore, 3 out of 139 (2.2%) infants from the APEHC cohort were infected with HIV-1 between January 2012 and October 2014 acquiring the virus through MTCT. Among the HIV-1-infected infants, one was an 8-month-old girl born in healthcare facilities at the end of 2012 who received oral zidovudine and formula feeding; her mother initiated ART (lamivudine/zidovudine/nevirapine) during the second trimester of pregnancy and received intrapartum zidovudine. The second infant was a one month old boy born at home in July 2014 who received formula feeding and his mother initiated ART (tenofovir/lamivudine/efavirenz) during the third trimester of pregnancy; no prophylaxis with zidovudine was administered. The third HIV-1-infected newborn was a disabled 7–month-old girl who was transferred to another hospital soon after delivery. No information on prophylaxis was available.

Finally, we calculated the cost per test using our assay, including cost of filter papers, reagents, equipment maintenance, and human resources to be 8-10 USD which is about 1/2 to 1/4 of the cost of commercialized tests in Angola. Hands-on time required to perform the assay was comparable based on information taken from company websites and references [24–26] Table 4.

**Table 4:** Comparison of cost and operational features of our in-house assay with commercial assays.

Features	In-house EID assay	AMPLICOR^TM^ HIV-1 DNA Test v1.5	Abbott RealTi*m*e HIV-1 Qualitative
Type of assay	PCR/qualitative	PCR/qualitative	Real Time PCR/Qualitative
Specimen volume	100-125 μl	200-500 μl	100-200 μl
Target of amplification	Pol (IN)	Gag	Pol (IN)
Genotypes detected	All subtypes, CRF02	Subtypes A-H	Subtypes A-H, CRF01, CRF02, Groups O and N
Analytical sensitivity (for DBS specimens)	112 copies/ml	Not available	839 copies/ml
Time for result	6-7 hours	7-8 hours	8 hours
Cost/test (USD)^*^	8-10	15-30	37
Number of samples/run	1-30 samples	9-21 samples	21-96 samples
Equipment required	Thermocycler, microcentrifuge, heat block, gel electrophoresis	Thermocycler, ELISA, reader/washer, microcentrifuge	M2000sp, M2000rt equipment
Equipment cost	14,000$	25,000$	150,000$

^*^Prices of tests performed with commercial assays vary considerably depending on quantities, infrastructure, support required and country of implementation.

## Discussion

Children are among the most vulnerable to be at risk for HIV infection but in spite of this the AIDS response in sub-Saharan Africa has largely left them behind [1]. In this regard, Angola has only registered a 25% reduction of new infections among infants since 2009 [1] which led the Assistant Secretary-General of the United Nations to declare in 2015 that the epidemic in Angola might worsen if an effective AIDS response is not reinvigorated. EID of HIV-1 infection in infants at risk enables early treatment and care of the infected infants. Significant progress has been made in many sub-Saharan countries in implementing EID services following the introduction of HIV DNA testing on DBS [27]. However, the high cost of commercially available HIV-1 DNA tests and the perceived sensitivity problems related to the very diverse and complex viral strains circulating in Angola have prevented their implementation in this country. At the HDP where our cohort is based and in most other hospitals in Angola, pediatric diagnosis of HIV-1 infection is still done by serology at month 12 of life which significantly delays the initiation of treatment [28]. To support EID service expansion in Luanda we developed and validated a new HIV-1 DNA assay to be used on DBS samples. To account for the very diverse HIV-1 strains present in Luanda, we had to produce a new set of control plasmids containing the IN gene, our target for amplification, from local HIV-1 subtypes. Phylogenetic analysis showed that most of the new IN sequences fall at basal positions on the phylogenetic trees (pre-subtype branches) which is consistent with the complexity of the HIV-1 strains circulating in Luanda and with Angola being one of the epicenters of the HIV-1 epidemic [10–12, 16–18].

To lower the costs, we used the Chelex method of DNA extraction which is also quick and easy to perform. This method had been previously applied to diagnostic screening in HIV-1-exposed infants in Rwanda showing reliable results when used in combination with either an in-house nested PCR or the Roche Amplicor HIV-1 DNA assay version 1.5 [29]. For the purpose of the EID, the Chelex DNA extraction method represents a low-cost alternative to commercial kits such as the QIAamp™ DNA Investigator Kit which costs 6.8 USD per reaction in Portugal and at least twice that in Angola. The Chelex method that we have used costs less than 3 USD per reaction, is very quick to perform, and does not use hazardous solvents. Moreover, a recent study that compared the yield of DNA extracted from blood samples applied to Whatman™ FTA™ cards using different methods showed that the amount of DNA recovered with the Chelex procedure (2.5 ng from a blood spot of 6.0 mm) is similar to or larger than the amount of DNA recovered with the other methods [30].

Our nested PCR assay showed a very low LoD for all the complex HIV-1 genotypes that we used as controls, suggesting that it was appropriate for early diagnosis of HIV-1 infection in infants in Angola. Indeed, using this assay we could detect all HIV-1 infected infants at month 1 of life. The good performance of the assay was also demonstrated in HIV-1 infected adults where we could detect HIV-1 DNA in 14.3% of patients with undetectable viral load (plasma viral load of <20 copies/mL).

The low percentage of HIV-1 infected infants (2.2%) in the APEHC cohort between 2012 and 2014 contrasts with the rate reported in 2014 at a national level of 25% [1] and confirms the effectiveness of the WHO-based prevention program implemented since 2007 at the HDP [28]. The detection of HIV-1 infection in infants as early as 1 month after birth makes this new assay suitable to health care centers following option B+ of WHO guidelines that recommend EID at 4-6 weeks of life. Moreover, our test might be useful to determine HIV-1 infection status when serology results are indeterminate after 12-18 months of age.

One possible shortcoming of our study is that we could not make a head-to-head comparison of our assay with a commercial test because currently there is no adequate platform for EID testing from DBS in Angola. In fact, the EID national program implemented at the *Instituto Nacional de Saúde Pública* (INSP) with the support of the Clinton Foundation was discontinued in 2012. NucliSens® HIV-1 QT test (bioMérieux, Inc., Durham, NC) is still used at the INLS but its performance is severely affected by the genetic heterogeneity of HIV-1. As reported by several studies, the test has low accuracy in detecting or quantifying specific group M subtypes (A, C, F, and G), recombinants (CRF01_AE, CRF02_AG), and group O [31–34]. Another possible shortcoming is the limited size of the prospectively enrolled cohort and consequently the clinical evaluation of the assay. Thus, further study in the clinical setting is likely warranted.

## Conclusions

The high analytical and clinical sensitivity of our EID assay have enabled accurate, early and low cost diagnosis of HIV-1 infection in exposed infants in Angola. The low percentage of HIV-1 MTCT case observed within the APEHC cohort is consistent with the current high standard of pediatric care provided at HDP. The simplicity and low-cost of the assay make it suitable for generalized implementation in Angola and other resource-constrained countries.

## Supporting information

S1 Fig Representative example of the results of the limit of detection (LoD) of the new PCR assay for subtype J control plasmid. This figure shows the LoD of the in-house assay determined using 500, 250 and 100 copies of subtype J control plasmid added to 125 ul of seronegative HIV blood and spotted in Human ID bloodstain cards.
(DOCX)

S2 Fig Results of the diagnostic specificity experiments using the new PCR assay with samples collected from adult patients infected with HBV, HCV or CMV. This figure show the results of the in-house assay when specimens of adults positive for common viruses were tested.
(DOCX)

S3 Fig Representative example of the results obtained using the new PCR assay on samples collected from infants enrolled in the APHEC cohort. This figure shows the results of the in-house assays when used for EID in HIV-1-exposed infants of our cohort.
(DOCX)

S1 Table Origin and genotype of the virus isolates used to produce the control plasmids. This table reports the origin, genotype and respective accession numbers of the virus isolates used to produce the control plasmids.
(DOCX)

S2 Table Sequence and location of PCR primers used in this study and size of amplified. This table reports the sequence and location of primers used for the amplification of the IN gene in clinical specimens, reference plasmids and ACH-2 cells.
(DOCX)

S3 Table Limit of detection (LoD) of HIV-1 subtype B DNA in ACH-2 cells using probit regression analysis. This table relates to the determination of the LoD of the in-house EID molecular test in ACH-2 cells using probit regression analysis. The same principle was applied to the control plasmids in order to determine the LoD for the different subtypes tested.
(DOCX)

S1 Data set file Data set underlying the findings in this study. 1) APEHC cohort dataset, collecting data of the pediatric population; 2) HIV-infected adults, positive controls attending the Hospital Egas Moniz; 3) HIV-infected infants, positive controls from the Angolan National Institute of Public Health.
(ZIP)
