# Supplementary material for: Early infant diagnosis of HIV-1 infection in Luanda, Angola, using a new DNA PCR assay and dried blood spots
Source: PLoS One. 2017 Jul 17;12(7):e0181352. doi: 10.1371/journal.pone.0181352 (PMC5513534; doi:10.1371/journal.pone.0181352)
Supplement: S1 Fig — This figure shows the LoD of the in-house assay determined using 500, 250 and 100 copies of subtype J control plasmid added to 125 ul of seronegative HIV blood and spotted in Human ID bloodstain cards. (DOCX) [file pone.0181352.s001.docx]

**S1 Fig.** **Representative example of the results of the limit of detection (LoD) of the new PCR assay for subtype J control plasmid**. 500, 250 and 100 copies of control plasmid were added to 125 ul of seronegative HIV blood and spotted in Human ID bloodstain cards. Extracted DNA was amplified by nested-PCR and amplified products were run on a 2% agarose gel with green safe staining. Each samples was amplified >10 times. (M) Molecular weight marker (NZY Leader VI); (IN) HIV-1 integrase fragment (194 bp); (R5) CCR5 gene fragment (189 bp); (SN) HIV-1 seronegative control; (-): ddH_2_O. The LoD for the subtype J was 4.3 copies/PCR (95% confidence interval: 3.3-6.3).

| 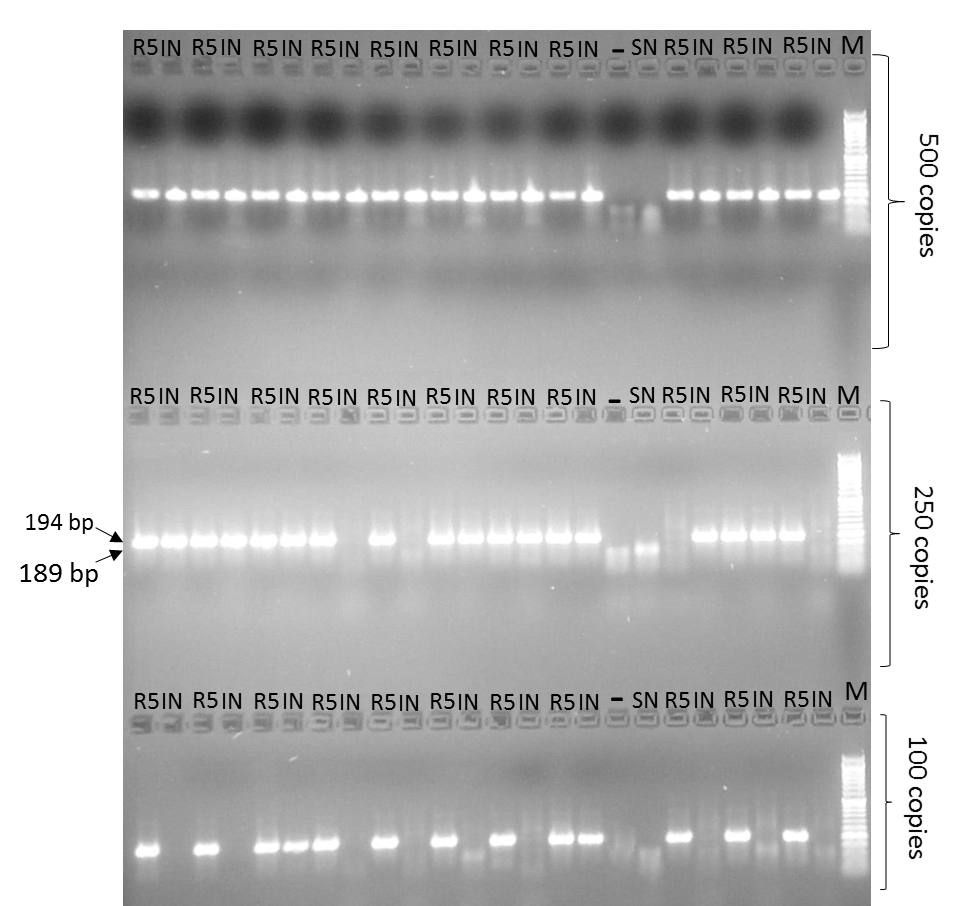 |
| --- |
