# Supplementary material for: Early infant diagnosis of HIV-1 infection in Luanda, Angola, using a new DNA PCR assay and dried blood spots
Source: PLoS One. 2017 Jul 17;12(7):e0181352. doi: 10.1371/journal.pone.0181352 (PMC5513534; doi:10.1371/journal.pone.0181352)
Supplement: S2 Fig — This figure show the results of the in-house assay when specimens of adults positive for common viruses were tested. (DOCX) [file pone.0181352.s002.docx]

**S2 Fig. Results of the diagnostic specificity experiments using the new PCR assay with samples collected from adult patients infected with HBV, HCV or CMV.** Amplification of samples from patients infected with HBV, HCV or CMV using the new PCR assay conditions. A) Samples of HBV-infected patients (n=10); B) Samples of HCV-infected patients (n=10); C) Samples of CMV-infected patients (n=10). Samples were tested in triplicate. PCR products were run on 2% agarose gel and stained with green safe. (M) Molecular weight marker (NZY Leader VI); (-) HIV-1 seronegative sample; (+) HIV-1 seropositive sample (194 bp); (R5) CCR5 gene (189 bp).

| 1A |
| --- |
| 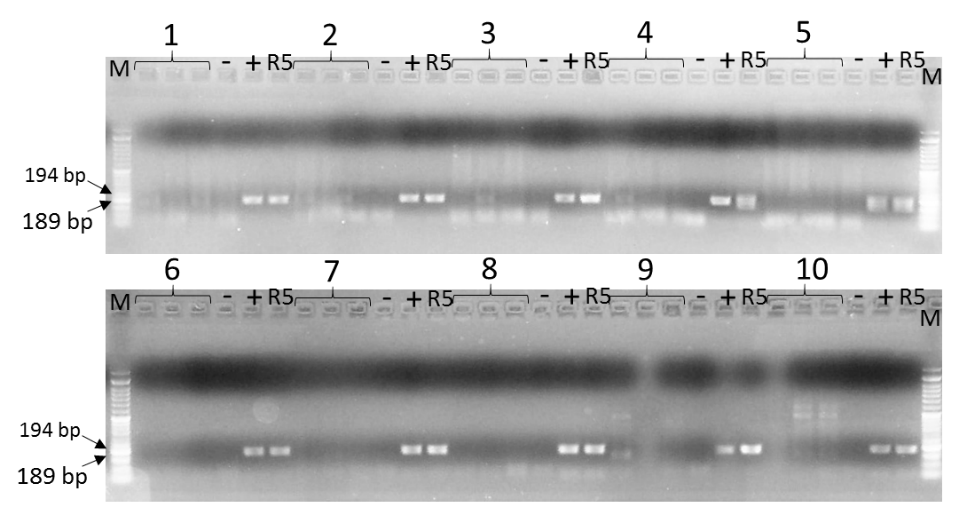 |
| 1B |
| 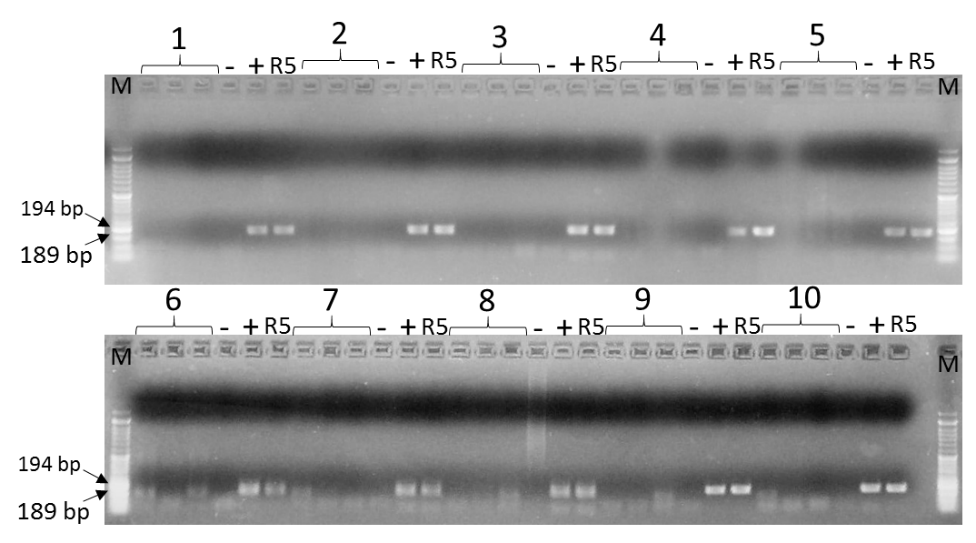 |
| 1C |
| 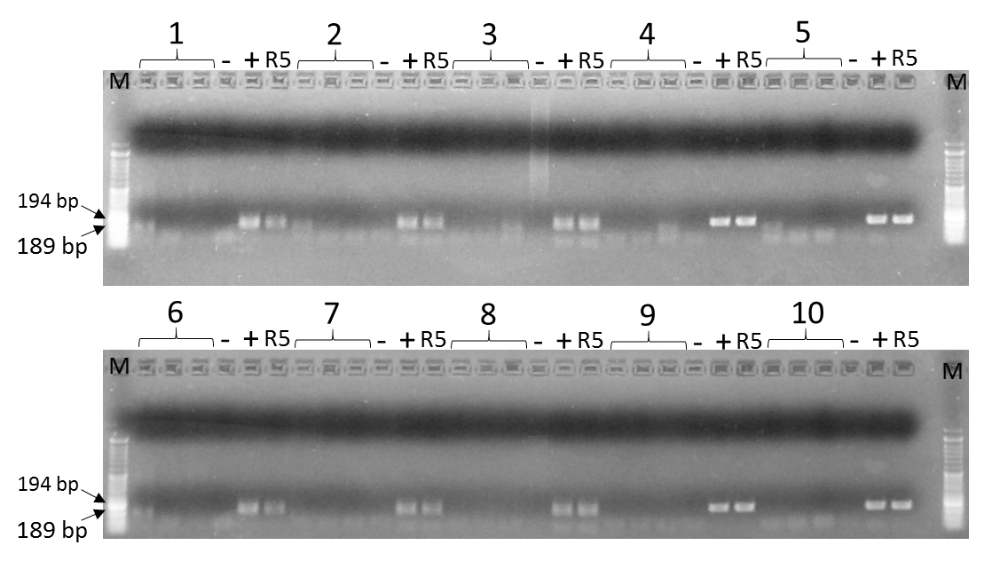 |
