# Supplementary material for: Early infant diagnosis of HIV-1 infection in Luanda, Angola, using a new DNA PCR assay and dried blood spots
Source: PLoS One. 2017 Jul 17;12(7):e0181352. doi: 10.1371/journal.pone.0181352 (PMC5513534; doi:10.1371/journal.pone.0181352)
Supplement: S3 Fig — This figure shows the results of the in-house assays when used for EID in HIV-1-exposed infants of our cohort. (DOCX) [file pone.0181352.s003.docx]

**S3 Fig. Representative example of the results obtained using the new PCR assay on samples collected from infants enrolled in the APHEC cohort.** Each infant was assigned an anonymized code (C126-C132). C126-C131 are uninfected infants whereas C132 is an HIV-1 infected infant. Samples were tested in triplicate. Amplified products were run on a 2% agarose gel with green safe staining. (M) Molecular weight marker (NZY Leader VI); (IN) HIV-1 integrase fragment (194 bp); (R5) CCR5 gene fragment (189 bp).

| 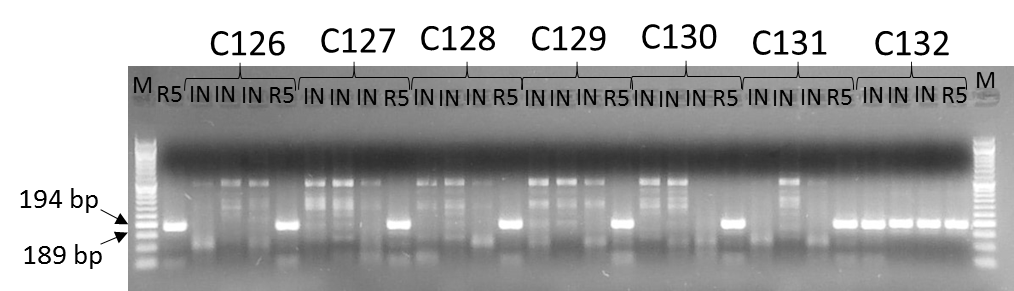 |
| --- |
