# Supplementary material for: Early infant diagnosis of HIV-1 infection in Luanda, Angola, using a new DNA PCR assay and dried blood spots
Source: PLoS One. 2017 Jul 17;12(7):e0181352. doi: 10.1371/journal.pone.0181352 (PMC5513534; doi:10.1371/journal.pone.0181352)
Supplement: S1 Table — This table reports the origin, genotype and respective accession numbers of the virus isolates used to produce the control plasmids. (DOCX) [file pone.0181352.s004.docx]

**S1 Table - Origin and genotype of the virus isolates used to produce the control plasmids.**

| **Isolate** | **Accession number** | **Sampling date** | **Country of Origin (Province)** | **Patient** | **Genotype**  **(IN gene)** |
| --- | --- | --- | --- | --- | --- |
| 93AOHDC249 | KU296949 | 1993 | Angola  (Cabinda) | adult | A1 |
| 93AOHDC251 | KU296950 | 1993 | Angola  (Cabinda) | adult | H |
| 93AOHDC253 | KU296951 | 1993 | Angola  (Cabinda) | adult | J |
| 09AOHDP34 | KU296952 | 2009 | Angola  (Luanda) | adult | C |
| 09AOHDP110 | KU296953 | 2009 | Angola  (Luanda) | adult | D |
| 09AOHDP157 | KU296954 | 2009 | Angola  (Luanda) | adult | G |
| 09AOHDP237 | KU296955 | 2009 | Angola  (Luanda) | adult | F1 |
| 01PTHDECJN | KU296956 | 1998 | Portugal* (Lisbon) | infant | CRF02_AG |
| 00PTHDEEBB | KU296957 | 2000 | Portugal* (Lisbon) | infant | G |

*Patients infected in Luanda
