# Supplementary material for: Early infant diagnosis of HIV-1 infection in Luanda, Angola, using a new DNA PCR assay and dried blood spots
Source: PLoS One. 2017 Jul 17;12(7):e0181352. doi: 10.1371/journal.pone.0181352 (PMC5513534; doi:10.1371/journal.pone.0181352)
Supplement: S2 Table — This table reports the sequence and location of primers used for the amplification of the IN gene in clinical specimens, reference plasmids and ACH-2 cells. (DOCX) [file pone.0181352.s005.docx]

**S2 Table- Sequence and location of PCR primers used in this study and size of amplified products.**

| **PCR type** | **Primers** | **Sequence (5’-3’)** | **Position in the HIV-1 HXB2** | **Band size (bp)** |
| --- | --- | --- | --- | --- |
| First-round PCR | F_IN_out | AACATAGTAACAGAYTCACARTATGC | 4,029-4,055 | 1,553 |
|  | R_IN_out | TGGTCTTCTGGGGCTTGTTCCAT | 5,582-5,559 |  |
| Second-round PCR | APEHC_IN_F | AATTGGAGAGCAATGGCTAGTGA | 4,281-4,303 | 194 |
|  | APEHC_IN_R | CACTGGCTACATGGACTGCTAC | 4,473-4,452 |  |
