# Supplementary material for: Early infant diagnosis of HIV-1 infection in Luanda, Angola, using a new DNA PCR assay and dried blood spots
Source: PLoS One. 2017 Jul 17;12(7):e0181352. doi: 10.1371/journal.pone.0181352 (PMC5513534; doi:10.1371/journal.pone.0181352)
Supplement: S3 Table — This table relates to the determination of the LoD of the in-house EID molecular test in ACH-2 cells using probit regression analysis. The same principle was applied to the control plasmids in order to determine the LoD for the different subtypes tested. (DOCX) [file pone.0181352.s006.docx]

**S3 Table - Limit of detection (LoD) of HIV-1 subtype B DNA in ACH-2 cells using probit regression analysis.**

| **Cells and provirus per DBS** | **Cells and provirus per PCR** | **No. Detected (%)** | **Probit value** |
| --- | --- | --- | --- |
| 5,000 | 50 | 10 (100) | NA ^*^ |
| 1,000 | 10 | 10 (100) | NA ^*^ |
| 500 | 5 | 8 (80) | 5.84 |
| 250 | 2.5 | 6 (60) | 5.25 |
| 100 | 1 | 3 (30) | 4.48 |
| 50 | 0.5 | 2 (20) | 4.16 |

^*^ NA, not applicable.
